# Effects of tranexamic acid on human nasal ciliary beat frequency

**DOI:** 10.1007/s00405-020-06602-7

**Published:** 2021-02-04

**Authors:** W. Behr, F. Horschke, A. Nastev, C. E. Mueller, J. U. Sommer, B. Folz, H. Li, U. W. Geisthoff, B. A. Stuck, R. Birk

**Affiliations:** 1 Department of Otorhinolaryngology, Head and Neck Surgery, University Hospital Marburg, Philipps-Universität Marburg, Baldinger Straße 1, 35043 Marburg, Germany; 2 Department of Otorhinolaryngology Head and Neck Surgery, University Hospital Wuppertal, Wuppertal, Germany

**Keywords:** Tranexamic acid, Epistaxis, Ciliary beat frequency, CBF, Ciliary dysfunction

## Abstract

**Background:**

Patients with recurrent epistaxis, particularly due to hereditary hemorrhagic telangiectasia (HHT) are recommended to apply topical tranexamic acid (TXA) to reduce bleeding events. Those patients may suffer ciliary dysfunction due to TXA’s effects on ciliary beating frequency (CBF) and their consequences.

**Methodology/principal:**

Human nasal epithelial cells were harvested with a nasal brush in 30 healthy subjects. We investigated the CBF in RPMI medium using high-frequency video microscopy. TXA was added to the cells in various concentrations ranging from 2 to 5%, including the therapeutic concentration (2%) and a control (0%).

**Results:**

CBF in the control condition was 6.1 ± 1.6 Hz. TXA reduces CBF in a time and concentration dependent manner, to, e.g. 4.3 ± 1.2 Hz with 2% TXA and 3.3 ± 0.9 Hz with 5% TXA after 16–20 min. The differences in CBF were statistically significant for all concentrations of TXA.

**Conclusions:**

TXA has the potential to significantly impair nasal epithelial function. Therefore, frequent or regular topical nasal application of TXA should be done under close monitoring of nasal function, especially in patients with co-morbidities like chronic rhinosinusitis.

## Introduction

The lifetime prevalence of epistaxis is 60%, and between 6 and 10% of affected patients need medical help. It is, therefore, an important condition that can cause a reduced quality of life [1]. The most common cause of epistaxis is trauma and mechanical irritation [1]. Epistaxis occurs more frequently in patients who are elderly, have co-morbidities like hypertension, are on antiplatelet therapy or have hereditary hemorrhagic telangiectasia (HHT). There are a few first aid measures recommended to patients to reduce the need of medical interventions and to support self-help, like applying pressure to the nose or nasal self-packing [2].

Local and systemic treatment with tranexamic acid (TXA) can also be used in the management of acute epistaxis. However, studies have shown inconclusive results in patients, regardless of whether they had HHT [3, 4]. Further preventative measures against recurrent epistaxis include application of nasal ointment or oil, nasal douching and avoidance of nose-blowing [5].

Even though there is no strong evidence of TXA’s effectiveness in comparison to other topical medications [6], patients with recurrent epistaxis are still recommended to apply local TXA to prevent serious bleedings [7] due to its pharmacological mode of action. TXA has a lysine-like structure and blocks the formation of plasmin. It reduces the proteolytic activity of plasminogen activators, inhibits fibrinolysis and is also used in other specialties in the management of bleeds [8]. Previous studies have shown that TXA minimizes acute bleeding complications when it was given orally or intravenously. Contraindications of systemic treatment include a history of thrombosis, breastfeeding, intracranial hemorrhage, renal dysfunction, history of seizures, hyperfibrinolytic conditions or allergy. Adverse effects like diarrhea, nausea and vomiting, seizures, headache, allergic dermatitis and vision problems also need to be considered [8]. Side- and adverse effects for topical application of TXA are either not known or considered as negligible [9]. However, there are contraindications for topical TXA usage in neurosurgery as it could trigger severe convulsive seizures when applied directly contact to the central nervous system [10]. TXA is widely available and accessible, is easy to apply and, therefore, represents a viable alternative treatment to reduce bleeding events, hospital admissions and to improve overall quality of life [2]. In a study with patients with HHT, a nasal ciliary dysfunction was observed, suggested to be an after-effect of the treatment [11]. Nasal ciliary epithelium and nasal beat frequency (CBF) are important in the mucociliary clearance and cleaning and defense mechanism of the nose. Factors that influence CBF vary and include temperature [12], pH [13] and tobacco [14]. Other topical medications like alpha-sympathomimetic drugs [15], estrogen [11], polyhexanide [16], and oils [17] are also shown to influence CBF. Up to now, there are no studies analyzing the effect of TXA on CBF of nasal human epithelial cells.

## Materials and methods

This study was carried out at the Department of Otorhinolaryngology, Head and Neck Surgery, University Hospital Marburg. The local ethics committee approved the study protocol (ref. number 117/19). Written informed consent was obtained from all participating subjects (15 female and 15 male). Exclusion criteria consisted of subjects under the age of 18, smokers, and subjects with chronic or acute upper airway disease and previous surgery of the nose. The measurement protocol was standardized according to the methodology of previous studies [16, 18]. Using a cytology brush, ciliary samples of the nose were harvested (Gynobrush Plus, Heinz Herenz, Hamburg, Germany). Standard procedure was to dip the brush in a 0.9% saline solution, before harvesting the cells via anterior rhinoscopy. Directly afterwards, a swap of each side of the nose was taken. Cell clusters were extracted out of middle and inferior nasal meatus by performing three full 360° rotations while pushing the brush from the anterior part of the middle meatus to the back of the inferior turbinate. The brush was removed out of the nose carefully and the cells were dipped into 5 ml of RPMI medium (RPMI 1640, cell culture tested, standard, L-glutamine: 300 mg/l, PromoCell, Heidelberg, Germany) with a slow rotary motion to dislodge the cells into the medium. Previous studies have shown that CBF of the ciliated epithelial cells remains stable between 3 and 9 h after harvesting [19] and external influences like temperature might impact CBF [20, 21]. Therefore, the medium was stored at 22 °C room temperature for 3 h.

With a pipette 1000 µl suspension of cells in RPMI were added in tissue culture plate (Sarstedt TC-Platte 6 Well Cell + , F. Sarstedt AG & Co. KG, Nümbrecht, Germany). The plates were scanned for vital cell clusters with a magnification up to 630-fold. CBF was examined for 20 min using an inverted phase contrast microscope (Leica DM IL LED Fluo, Leica Microsystems GmbH, Wetzlar, Germany) and a high-speed video camera (BASLER acA 1300-200um), recording at 200 frames per second (fps). Video sequences with a duration of 2 sec were recorded in 2-min intervals. Using the Sissons-Ammons-Video-Analysis (SAVA) software (Ammons Engineering, 8114 Flintlock Road, Mt. Morris, MI 48458, USA) the ciliary frequency of beating cells was measured three times in every sequence and the mean average CBF of the measurements was determined [22]. To analyse the CBF, the region of interest (ROI) method was selected by a blinded observer. Three ROIs were selected in each video sequence and the mean average CBF was calculated by the software. Observations for the medium without additional solutions were stopped after 20 min, as no changes were seen within this time span. This group served as the negative control group.

To evaluate the effects of TXA, 500 µl of the harvested RPMI cell solution and 500 µl of 10% TXA-solution (Tranexamsäure 100 mg/ml, CARINOPHARM GmbH, Elze, Germany) were mixed, to form a TXA concentration of 5%. The same procedure was followed as described above for a time span of 30 min to determine the mean average CBF.

200 µl of 10% TXA-solution was added to 800 µl of the cell-suspension to examine behavior of cilia in a 2% dilution. The same procedure to determine ROI was repeated for the 2% solutions. The TXA used in this study contained TXA, water and hydrochloric acid to adjust pH value. According to the technical information, the pH value of the TXA solution used lies between 6.5 and 7.5. To mitigate dilutional effects on ciliary beat frequency, water was tested in a separate measurement as a control. No significant changes in CBF were seen in the dilution of the RPMI-cell mixture and water 1:1 for 20 min. Statistical analysis was completed via Mann–Whitney *U* test using the open source program “R” [23].

## Results

All recorded video sequences were analyzed successfully. Calculations showed a reduction of CBF with increasing concentrations of TXA. The mean average CBF measured over the entire period was 6.2 ± 1.6 Hz in the control group (Table 1). In 2% TXA solution, the mean average CBF was 4.4 ± 1.3 Hz. In 5% TXA solution, there was a mean average CBF of 3.4 ± 0.9 Hz (Table 1).

**Table 1:** Ciliary beat frequency (CBF) in RPMI 1640, cell culture tested, standard, l-glutamine: 300 mg/L, PromoCell, Heidelberg, Germany) and 2% and 5% suspension of tranexamic acid (TXA)

Negative control		TXA 2%		TXA 5%	
Time (min)	CBF	Time (min)	CBF	Time (min)	CBF
2	6.7 ± 1.4 Hz	2	5.3 ± 1.6 Hz	2	3.9 ± 1.0 Hz
6	6.2 ± 1.6 Hz	6	4.7 ± 1.2 Hz	6	3.6 ± 0.9 Hz
12	6.2 ± 1.6 Hz	12	4.2 ± 1.1 Hz	12	3.4 ± 1.0 Hz
16–20	6.1 ± 1.6 Hz	16–20	4.3 ± 1.2 Hz	16–20	3.3 ± 0.9 Hz

**Fig. 1:**
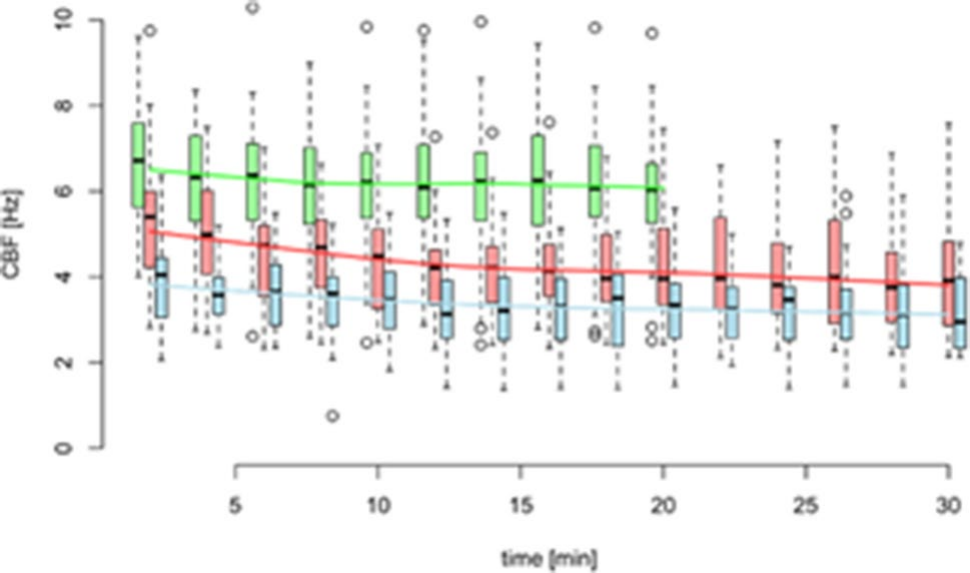
Boxplot of ciliary beat frequency (CBF) in concentrate with 0% (green), 2% (red) and 5% (blue) concentrate of TXA. The boxes show interquartile range (IQR) with whiskers extending up to 1.5 times the IQR. The solid line marks the median. CBF in Hz, time in min. *N *= 30

**Fig. 2:**
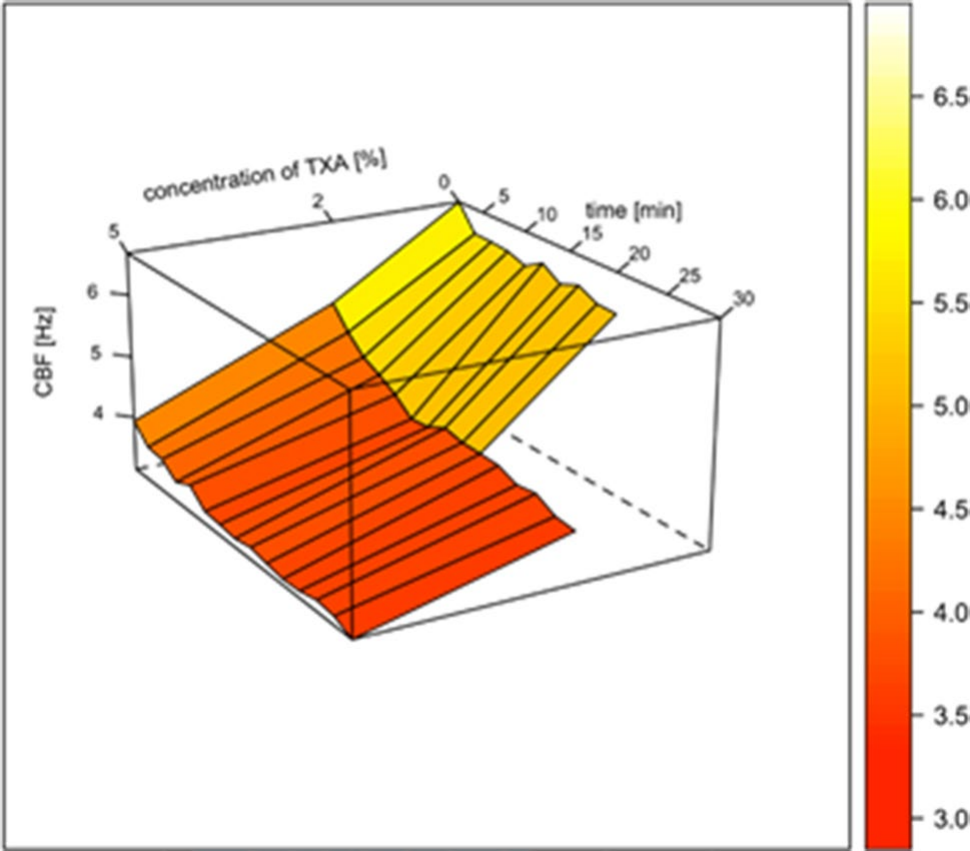
3D diagram of time- and concentration-dependent changes of ciliary beat frequency (CBF) by TXA in vitro. CBF in Hz, time in min. *N* = 30

The difference of the average mean CBF in both TXA concentration groups of 2% and 5% were statistically significant compared to the control group at each point of time. For example, the *p* value for the difference of the mean CBF of the control group versus TXA 2% at *t* = 2 min was < 0.0001. These highly significant results extend over the entire measurement period for both concentrations. Graphical interpretation is shown in Figs. 1 and 2 in a box-and-whisker plot and a three-dimensional diagram.

## Discussion

Studies have shown a reduced CBF in patients with chronic rhinosinusitis or rhinitis [24, 25], as well as chronic obstructive pulmonary disease (COPD) [26]. Recent studies suggest that patients with HHT may also have a reduced CBF [11]. The results in this study show that TXA reduces CBF significantly in vitro and consequently, may reduce mucociliary clearance in vivo. This is clinically relevant, as impaired mucociliary clearance can impair respiratory defense mechanisms and consequently lead to biofilm formations and relapsing and chronic infections [27].

Bleeding events are the most common presenting complaint in patients with HHT. Patients suffering from recurrent epistaxis need a multimodal management plan, including self-administered treatments [28], as discussed above.

To draw conclusions regarding everyday clinical practice, a distinction should be made between acute treatment and long-term therapy with TXA. The use of topical TXA is a common treatment option in the management of acute epistaxis and is supported by some studies with positive results [29, 30]. It is less invasive and better tolerated than nasal packing or coagulation, which is often painful for patients [31]. The length of hospital stay in patients who were managed with topical TXA compared to patients who received standard care of epistaxis was also not found to be significantly statistically different [3].

Quality of life increases with less hospitalizations [2]. A systematic review has shown that topical TXA reduces bleeding events and the need for blood transfusions in general surgery [4]. Self-management options in epistaxis, like topical application of TXA or nasal self-packing, can also increase the perceived quality of life [6]. However, there are yet no studies on self-administered topical TXA ointment and their effects on preventing epistaxis episodes. The only existing study has relevant limitations, as the application form was not standardized [6].

Our data indicate that frequent nasal application of TXA should be provided with caution as prophylaxis for epistaxis, especially when the patient has other comorbidities like chronic rhinosinusitis. Patients with HHT usually undergo multiple surgical interventions in the nose. As a result, the anatomy and physiology are often altered with scars and damaged mucosal membranes. Furthermore, it is suggested that patients with HHT may have reduced CBF [11] and may have other nasal anomalies like respiratory epithelial adenomatoid hamartoma (REAH) [32].

A possible cause for the reduced CBF may be the change in pH value of the ciliated cells. Previous studies have shown that acidic pH values under 7 significantly reduces the CBF of bronchiolar ciliated cells [33]. Another group demonstrated that nasal saline irrigations with different pH values have varying effects on nasal symptoms and presumably has an impact on mucociliary clearance [13]. The pH value of the TXA in this study was between 6.5 and 7.5. Physiological pH in the nose is 6.8 [34].

The risk of additional mucociliary dysfunction must be considered and patients should be informed. Based on our data, we recommend that patients with a pre-existing chronic inflammation of the upper airways should be closely monitored by an ENT specialist. If there have been no bleeding events for several months, the use of topical TXA may be considered to be reduced or stopped. The risks and benefits should be weighed carefully, especially in patients with HHT. If recurrent epistaxis cannot be controlled conservatively, surgical interventions like nasal occlusion should be considered in an experienced center [35]. Long-term use of TXA in HHT patients could possibly further damage already strained nasal mucosa and consequently lead to biofilm formation with bacterial colonization, chronic inflammation and nasal congestion. This would significantly affect the normal physiological tasks of the nose, such as warming and humidification of air [36].

### Limitations of the study

The present study is an in vitro study without examination of the long-term use of TXA and, therefore, only allows to draw limited conclusions for the clinical practice. In addition, conclusions regarding mucociliary clearance in vivo, especially in patients with in HHT with potentially damaged mucosa, can only be drawn to a limited extent. Another limitation includes the delay of 1–2 min between the application of TXA and the first measurement of CBF as this time was required for the identification of a beating cell cluster. Consequently, we are unable to determine the speed of TXA’s effects on CBF until the first video sequence. However, the effects of the delay were minimized by taking measurements for at least 20 min. The in vivo CBF and the impact of external influences may also be underestimated in this study. Possible effects of other ingredients of established TXA mixture (e.g. ointment: 1,5 g tranexamic acid, 25 g paraffinum (sub)liquidum, 25 g lanolin, 35 g Unguentum Cordes ^®^) and the reversibility of CBF have not been assessed.

## Conclusion

The aim of this study was to evaluate the impact of TXA on CBF. The results show that TXA reduces CBF significantly. Therefore, long-term use of TXA to prevent epistaxis should be considered with care.

## Data Availability

Information about data and material is given in the paper and references.
